# 数字化胸腔引流系统用于肺部手术后难治性持续性漏气的疗效观察

**DOI:** 10.3779/j.issn.1009-3419.2017.12.07

**Published:** 2017-12-20

**Authors:** 东来 陈, 西尔买买提·卡德尔, 哲 施, 宇星 金, 昶 陈

**Affiliations:** 200433 上海，同济大学附属上海市肺科医院 Department of Thoracic Surgery, Shanghai Pulmonary Hospital, Tongji University School of Medicine, Shanghai 200433, China

**Keywords:** 数字化胸腔引流系统, 难治性持续性漏气, 肺切除, 外科, Digital drainage system, Postoperative refractory prolonged air leaks, Pulmonary resection, Surgery

## Abstract

**背景与目的:**

术后持续性肺漏气是肺部手术后最常见的并发症之一。数字化胸腔引流系统（digital drainage system, DDS）被认为具有准确、客观、舒适、患者耐受性好、经济等优点。本研究旨在总结DDS用于肺部手术后难治性持续性大量漏气的临床疗效。

**方法:**

术后5天和7天分别是胸腔镜和开胸术后持续性肺漏气（prolonged air leak, PAL）的界限；本研究将难治性持续性大量漏气定义为达到PAL时间2倍及以上且漏气持续达2度以上，可伴有明显皮下气肿或纵隔气肿等相关并发症。2016年1月-2016年12月间，应用数字化胸腔引流系统结合胸膜固定术治疗符合上述标准的病例共8例，收集相关临床资料并总结分析。

**结果:**

本组病例中肺叶切除术6例，肺段切除2例。平均术后持续漏气（17.3±5.1）d，DDS平均使用时间（5.6±3.7）d。平均术后引流量（3, 550.6±1, 881.1）mL。应用DDS前后的平均引流量为（2, 615.6±1, 741.2）mL和（935.0±242.7）mL。平均住院时间（18.1±5.0）d。

**结论:**

数字化胸腔引流系统用于肺部手术后难治性持续性大量漏气安全可行，可提供更大的负压吸引值，从而促使患肺复张为后继的胸膜固定术创造条件，并缩短引流天数和住院时间。

## Abstract (en)

**Background and objective:**

Postoperative prolonged air leaks is one of the most common complications secondary to pulmonary resections. Digital drainage system (DDS) is considered as an accurate and objective device which has been found to be comfortable and well tolerated by patients, economical as well. The aim of this study is to explore the application of DDS in patients with postoperative refractory prolonged air leaks after pulmonary surgery.

**Methods:**

Prolonged air leak (PAL) is defined as air leaks lasting for more than 5 d after video-assisted surgery or more than 7 d after thoracotomy. Postoperative refractory prolonged air leak is defined as a kind of air leak lasting for twice than PAL with grade 2 or 3 air leak, or air leak with severe complications such as subcutaneous or mediastinal emphysema in our study. A total of 8 patients who had postoperative refractory prolonged air leaks after pulmonary resection were treated with digital drainage system combined with pleurodesis from January to December in 2016 in Department of Thoracic Surgery, Shanghai Pulmonary Hospital. All the relevant clinical data of patients were collected for analysis.

**Results:**

In our study, 6 patients accepted lobectomy, the other 2 patients underwent segmentectomy. The average air leak and chest tube duration of the patients with postoperative refractory prolonged air leaks after pulmonary resection was (17.3±5.1) d. The average postoperative DDS duration was (5.6±3.7) d. The mean drainage volume of the patients was (2, 615.6±1, 741.2) mL and (935.0±242.7) mL before and after the application of DDS. The average length of hospital stay was (18.1±5.0) d.

**Conclusion:**

It is safe and feasible to apply digital drainage system to patients with postoperative refractory prolonged air leaks after pulmonary resection. Compared with the conventional drainage system, digital drainage system is more accurate and objective which can offer a larger suction pressure and promote lung recruitment maneuver for pleurodesis, shortening the drainage time and length of hospital stay.

术后肺漏气是肺部手术后最常见的并发症之一^[1]^，高达50%以上的患者在术后初期存在漏气。通常认为，一旦肺漏气时间超过术后平均住院日，即可称为持续性肺漏气（prolonged air leak, PAL）^[2]^。美国胸外科医师协会（Society of Thoracic Surgeons）和欧洲胸外科医师协会（European Society of Thoracic Surgeons）数据库都以持续肺漏气时间长于5 d为PAL的界定标准^[3-5]^。Fobury和Naunheim等则认为该标准受医疗水平、住院时间等多项影响因素而变化，难以确切界定，并大致认为开胸手术者为7 d，胸腔镜手术者为5 d，逾期为延迟性肺漏气^[6]^。据欧洲胸外科医师协会数据库统计，肺叶、肺段和楔形切除术后长于5 d的PAL发生率分别为8.6%、6.7%和3.5%^[7]^。术后PAL与难治性持续性大量漏气对患者有诸多不利影响，主要为增加其他并发症的风险^[8]^、延长引流时间和住院时间^[1, 8, 9]^，这无疑也增加了患者的精神和经济负担。

传统的引流系统通常由胸腔引流管和水封瓶组成，最大可提供16 cmH_2_O的负压，但这种引流装置既限制术后患者的活动^[10, 11]^，同时也由于水封瓶中的气泡产生较大噪音影响患者休息。对于手术后持续性大量漏气和患肺不能完全复张与胸腔匹配的情况，临床上多采用鼓励咳嗽和吹气球促使患肺膨胀的方法，从而为后继的胸膜固定创造条件；或者以等待胸内残腔自行固定的方法达到拔管条件。然而上述方法都存在耗时长、带管期间患者生活质量严重受限等缺点。本研究组针对上述情况，尝试利用数字化引流系统（digital drainage system, DDS）的超大负压吸引值（30 cmH_2_O）、可视化图表和定量检测记录功能^[9-13]^，解决上述临床难题。现将初步经验报道如下。

## 1 材料和方法

### 1.1 病例选择

本研究以长于5 d和7 d作为胸腔镜和开胸手术术后PAL的界限，并将难治性持续性大量漏气定义为达到PAL时间2倍及以上且漏气达2度以上的肺漏气，伴有或不伴有皮下气肿或纵隔气肿等相关并发症。我院胸外科于2016年1月-2016年12月期间应用DDS进行术后引流20例，其中包括符合上述标准的难治性持续性大量漏气共8例。

### 1.2 数字化引流系统

本研究采用的数字化胸腔引流系统为速衡Thopaz，由Medela医疗科技公司提供。该装置具有记录患者编号，调整并记录吸引负压、引流速度的功能，同时在引流瓶已满、系统泄露、系统阻塞、自检失败、引流瓶中滤菌器阻塞和系统过热或电池耗尽时均可发出警报，并可在显示屏上反馈当前问题。

### 1.3 PAL的治疗流程

对术后存在PAL的患者，根据患肺复张的程度决定胸膜固定的时机。若患者并发明显皮下气肿或纵膈气肿，须在确定引流通畅的同时，观察气肿的吸收情况。一旦气肿大致吸收，且医师通过胸部平片可观察到患肺复张与胸壁接触贴合，则可尝试行胸膜固定。对于不确定患肺是否和胸壁贴合者，可以进行CT扫描确认，同时可观察胸腔引流管的确切位置。胸膜固定通常采用50%的高渗糖进行灌注，在灌注后予以不同体位摇动患者，促使大范围的粘连形成。上述步骤重复2 d-3 d后通常可观察到漏气减少，伺漏气消失再复查胸片并拔除引流管。数字化引流系统启用后，根据引流效果及时调整负压参数。在确定上胸管处于胸顶的情况下，将负压调整到患者能耐受的最大值。

### 1.4 统计学分析

收集所有病例的相关临床资料，包括手术方式、引流时间、引流量、术后胸膜固定时机和次数、使用DDS的起止时间、术后并发症和住院时间等。应用统计学软件（SPSS 17.0统计软件）行数据分析描述。

## 2 结果

### 2.1 一般情况

所有8例术后难治性持续性大量漏气患者中，男5例，女性3例。左侧引流2例，右侧4例，双侧引流2例（表 1）。8例患者术前均有不同程度的肺气肿或肺大疱，其中2例患者既往有结核病史。

**1:** 接受肺部手术后出现难治性持续性漏气的8例患者的临床资料 Clinical information of 8 patients with postoperative refractory prolonged air leaks after pulmonary surgery

Age	Gender	Surgical sites	Surgical procedures	Postoperative pathological results	Hospital stay
62	M	LUL	Lob v	Invasive adenocarcinoma	24
72	M	RUL	Lob	Invasive mucinous adenocarcinoma	21
70	M	RUL	Lob v	Fibrous tissue accompanied with collagen and carbon deposition	19
64	M	RUL	Seg v	Atypical adenomatous hyperplasia	22
		LUL	Seg v	Atypical adenomatous hyperplasia	
48	F	RUL	Wed v	Atypical adenomatous hyperplasia	12
		LUL	Lob v	Adenocarcinoma *in situ*	
		LLL	Wed v	Minimally invasive adenocarcinoma	
72	M	LUL	Lob v	Invasive adenocarcinoma	12
75	F	RLL	Lob v	Invasive adenocarcinoma	13
71	F	RLL	Lob v	Invasive adenocarcinoma	22
LUL: left lower lobe; RUL: right upper lobe; LLL: left lower lobe; RLL: right lower lobe. Lob v: lobectomy by VATS (video-assisted thoracoscopic surgery); Seg v: segmentectomy by VATS; Wed v: wedge resection by VATS; M: male; F: female.					

### 2.2 术后并发症

8例术后难治性持续性漏气患者中，使用DDS前有肺不张2例，轻度肺部感染4例，于出院时均已治愈，平均住院时间（18.1±5.0）d（表 1）。

### 2.3 与漏气相关的信息

本组患者术后持续漏气/引流天数平均（17.3±5.1）d，应用DDS时间平均（5.6±3.7）d，平均住院天数（18.1±5.0）d，平均术后总引流量（3, 550.6±1, 881.1）mL，应用DDS前后的平均引流量为（2, 615.6±1, 741.2）mL和（935.0±242.7）mL（表 2）。

**2:** 使用数字化胸腔引流系统的相关信息 Relevant information of using DDS

Serial number	Overall drainage period (d)	Postoperative drainage volume (mL)	Start date of using DDS (d)	Duration on DDS (d)	Drainage volume before DDS (mL)	Drainage volume during DDS (mL)	Date to perform pleurodesis (d)
1	25	7, 630	22	3	6, 530	1, 100	20
2	19	2, 730	12	7	2, 000	730	10
3	19	3, 025	10	9	2, 025	1, 000	7
4	22	1, 500	10	12	690	810	
5	12	2, 730	10	2	2, 030	700	
6	12	2, 830	10	2	2, 100	730	8
7	13	3, 140	10	3	2, 120	1, 020	8
8	22	4, 820	15	7	3, 410	1, 410	13

## 3 讨论

Pompili等^[7]^根据是否存在外部吸引将胸腔引流系统分为被动和主动引流，同时根据能否自动调节吸引水平将系统分为可调节吸引型和固定吸引型。传统的胸腔引流系统属于固定吸引型的被动引流系统，而DDS属于可调节吸引型的主动引流系统。后者作为一种新型的引流方式近年来在胸外科领域被广泛应用，并得到了许多临床医生的报道肯定，其主要优势如下：①可对患者漏气情况进行准确评估，对观察者而言具有可重复性和客观性；②实时监测将漏气过程图像化数据化，可及时反馈导管阻塞或分离、吸引无效等情况，从而有助于临床医生判断当前引流的有效性；③吸引负压和引流速度的可调节性大，并为临床医生决定拔管时机提供了客观依据；④携带便捷且不影响患者活动；⑤持续大量漏气患者可携带该系统在家中引流并将相关数据向医生报告，从而缩短了住院时间。根据我院8例难治性持续性漏气患者的术后引流数据来看，上述优势亦有体现，使用DDS期间患者舒适度更高、耐受更好，有利于临床医生更高效地进行术后管理。

肺切除术后肺体积减小，同侧胸膜腔容积相对增大，两者的适配过程直接影响到手术后肺漏气的持续时间长短。据文献^[3, 7, 14]^报道，PAL的风险因素包括肺实质受损或呼吸系统疾病引起的肺功能降低（FEV_1_＜80%）、肺上叶切除、身体质量指数（body mass index, BMI）不足（BMI＜25.5）和胸膜粘连，且年龄＞65岁、性别为男性、长期应用类固醇类药物、合并慢性阻塞性肺疾病（chronic obstructive pulmonary disease, COPD）、糖尿病的患者术后发生PAL的风险亦增高^[15]^。既往对于难治性持续性大量漏气患者，通常采用让患者带管出院继续引流，待患者发生胸膜增厚、膈肌上抬、纵隔偏移、余肺代偿性扩张等解剖形态变化并实现充分的解剖形变匹配，进而消除漏气，该过程通常需要数周，甚至数月。本研究为国内首次报道的将DDS应用于肺部术后难治性持续性大量漏气患者，本组的8例肺部手术后发生的难治性持续性漏气患者，通过使用DDS进行负压吸引引流后均于较短时间内实现拔管和出院，为患者减轻了经济负担和精神负担。由于出现难治性持续性漏气的患者总数较少，因此本研究仅能采用单臂试验，对照既往临床经验论证其可行性和优越性，这是本研究存在的不足之处，但根据使用DDS引流前后的数据对比和总引流天数等相关资料仍能反映出DDS主动高负压吸引引流系统在此类病例中应用的优势。结合现有的DDS和传统引流方法对术后漏气引流效果对比的随机对照临床研究（表 3），我们有较为充分的证据说明将DDS用于难治性持续性大量漏气患者的引流是有临床参考价值的。

**3:** 数字化系统和传统引流方式在漏气患者中的疗效对比 Digital system versus traditional system in patients with air leak

Study	Air leak duration (d)	Chest tube duration (d)	Hospital stay (d)
Cerfolio and Bryant, 2009^[1]^	NA	3.0 *vs* 4.4^*^	3.9 *vs* 4.6
Mier *et al*, 2010^[1]^	NA	2.4 *vs* 4.5^*^	NA
Pompili *et al*, 2014^[7]^	1.0 *vs* 2.2^*^	3.6 *vs* 4.7^*^	4.6 *vs* 5.6^*^
Gilbert *et al*, 2015^[1]^	NA	4.9 *vs* 5.6	6.2 *vs* 6.2
Filosso *et al*, 2016^[16]^	NA	3.0 *vs* 4.0^*^	7.0 *vs* 8.0^*^
Miller *et al*, 2016^[1]^	2.7 *vs* 3.9^*^	3.7 *vs* 4.3^*^	4.1 *vs* 5.6^*^
Shoji *et al*, 2016^[9]^	NA	2.7 *vs* 3.7^*^	NA
NA: not available; ^*^: Differences are statistically significant.			

综上，我们认为数字化胸腔引流系统对肺部手术后难治性持续性漏气患者的治疗效果是令人满意的。其在临床应用中具有安全性和可行性，患者耐受性良好，与传统引流系统相比更加客观准确，可提供更大的负压吸引值，可缩短引流天数和住院时间从而降低患者经济负担，值得在有条件的单位和患者中尝试应用。
